# SpliceWiz: interactive analysis and visualization of alternative splicing in R

**DOI:** 10.1093/bib/bbad468

**Published:** 2023-12-27

**Authors:** Alex C H Wong, Justin J-L Wong, John E J Rasko, Ulf Schmitz

**Affiliations:** Gene and Stem Cell Therapy Program, Centenary Institute, the University of Sydney, Camperdown, NSW 2050, Australia; Epigenetics and RNA Biology Laboratory, School of Medical Sciences, Faculty of Medicine and Health, the University of Sydney, Camperdown, NSW 2050, Australia; Faculty of Medicine and Health, the University of Sydney, Camperdown, NSW 2050, Australia; Epigenetics and RNA Biology Laboratory, School of Medical Sciences, Faculty of Medicine and Health, the University of Sydney, Camperdown, NSW 2050, Australia; Faculty of Medicine and Health, the University of Sydney, Camperdown, NSW 2050, Australia; Gene and Stem Cell Therapy Program, Centenary Institute, the University of Sydney, Camperdown, NSW 2050, Australia; Faculty of Medicine and Health, the University of Sydney, Camperdown, NSW 2050, Australia; Cell and Molecular Therapies, Royal Prince Alfred Hospital, Camperdown, NSW 2050, Australia; Biomedical Sciences and Molecular Biology, James Cook University, Townsville, QLD 4810, Australia; Centre for Tropical Bioinformatics and Molecular Biology, James Cook University, Townsville, QLD 4810, Australia

**Keywords:** differential splicing, intron retention, exon usage, read coverage, gene isoforms, data visualization

## Abstract

Alternative splicing (AS) is a crucial mechanism for regulating gene expression and isoform diversity in eukaryotes. However, the analysis and visualization of AS events from RNA sequencing data remains challenging. Most tools require a certain level of computer literacy and the available means of visualizing AS events, such as coverage and sashimi plots, have limitations and can be misleading. To address these issues, we present *SpliceWiz*, an R package with an interactive Shiny interface that allows easy and efficient AS analysis and visualization at scale. A novel normalization algorithm is implemented to aggregate splicing levels within sample groups, thereby allowing group differences in splicing levels to be accurately visualized. The tool also offers downstream gene ontology enrichment analysis, highlighting ASEs belonging to functional pathways of interest. *SpliceWiz* is optimized for speed and efficiency and introduces a new file format for coverage data storage that is more efficient than BigWig. Alignment files are processed orders of magnitude faster than other R-based AS analysis tools and on par with command-line tools. Overall, *SpliceWiz* streamlines AS analysis, enabling reliable identification of functionally relevant AS events for further characterization. *SpliceWiz* is a Bioconductor package and is also available on GitHub (https://github.com/alexchwong/SpliceWiz).

## Introduction

Alternative splicing (AS) is a regulated process whereby alternate blocks of protein coding information, exons, are selectively transferred during or after transcription of messenger RNA from DNA. Introns, sequences interspersed between exons, are removed by the spliceosome at their donor (5′) and acceptor (3′) splice sites. AS arises consequent to enhanced or inhibited recognition of splice sites [1, 2], leading to selective retention or removal of extra genetic information during or after transcription. Although AS can be complex, at its essence, there are seven basic forms [3], namely inclusion/skipping of cassette exons (skipped exons, SE), inclusion of mutually exclusive exons (MXE), alternative 5′/3′ splice site usage (A5SS/A3SS), alternative first or last exons (AFE/ALE) and intron retention (IR) (Figure S1). These alternative splicing events (ASEs) are defined as a binary choice between ‘inclusion’ or ‘exclusion’ of the alternate exon, partial exon or intron. ASEs can be quantified using RNA sequencing reads aligned across alternate splice sites (hereafter junction reads) [4], and are visualized using coverage or sashimi plots [5]. AS allows a single gene to give rise to multiple messenger RNA transcripts from the same DNA template, which in turn can translate different proteins [6]. Additionally, retained introns can affect transcript localization and degradation, leading to altered expression [7, 8].

Despite the ready availability of RNA sequencing, including a vast volume of publicly available datasets, AS analysis is not routinely performed. Most tools for AS analysis are command-line based, hindering their accessibility to those not familiar with such interfaces. Coverage and sashimi plot visualization is limited by the lack of methods to normalize coverage at genomic regions of ASEs. If such methods existed, differential splicing between conditions could be visualized using mean normalized coverage of replicates within each condition. Individual samples from each condition with large differences in coverage can often be found even in ASEs with no significant differential splicing between conditions. Thus, the current practice of selecting ‘representative individual samples’ is flawed and leads to publication bias. Finally, data interactivity is lacking in current tools, particularly downstream to differential splicing analysis. Interactive data exploration tools would facilitate the process of identifying functionally relevant ASEs for further study.

To address the above limitations, we created *SpliceWiz*, an AS analysis and visualization tool. *SpliceWiz* is implemented as an R package and provides both graphical and command-line user interfaces. Starting from input bulk RNA sequencing alignment files (BAM files), *SpliceWiz* quantifies AS using junction reads and intronic coverage, leveraging novel junction reads to detect cryptic splice sites and exons. Differential analysis is performed using established statistical tools available in R, allowing generalized linear model (GLM) based analysis to accommodate large complex datasets. We propose a coverage normalization method to visualize IR ratios, and show that this approach accurately visualizes differential AS. A variety of interactive visualization tools are implemented, including gene ontology (GO) enrichment analysis, to facilitate identification of reliable candidate ASEs relevant to the observed biological phenomena. Finally, to improve computational performance we optimized storage and retrieval of sequencing coverage using a new file format, implemented alongside multi-threaded parsing of alignment files. Taken together, *SpliceWiz* is an accessible, user-friendly and computationally efficient application that streamlines AS analysis, focussing on reliably identifying functionally relevant ASEs for further characterization.

## Results

### The *SpliceWiz* pipeline

**Figure 1:**
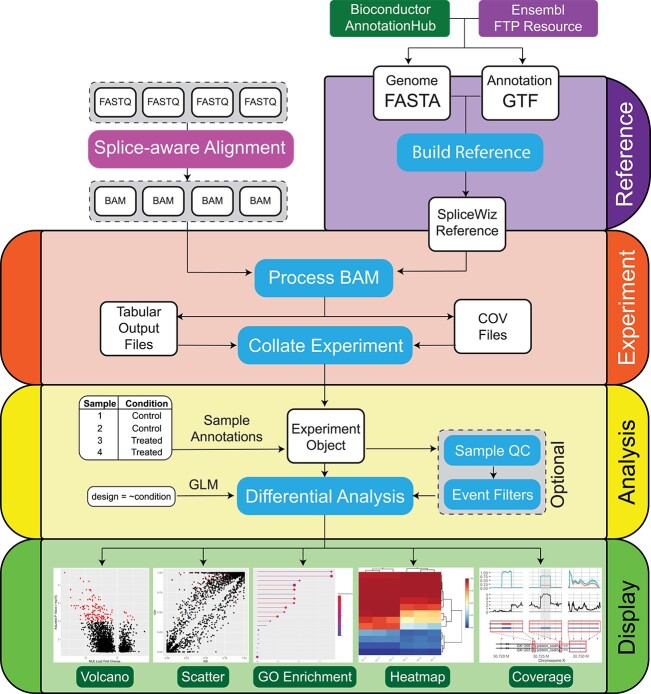
The SpliceWiz pipeline is organized into four modules. ‘Reference’ creates the SpliceWiz alternative splicing annotations required for processing alignments. ‘Experiment’ contains functions to process alignment files and collate their outputs into a dataset. ‘Analyse’ imports this dataset, allowing users to review quality control metrics and perform pre-test filtering of lowly-represented ASEs, and performs differential analysis to generate a table of results. ‘Display’ provides visualization tools, including volcano and scatter plots, gene ontology enrichment analysis, heatmaps and coverage plots.

We implemented *SpliceWiz* as an R package, with both a command-line and graphical interface, the latter implemented via *shinyDashboard*. We organized the *SpliceWiz* pipeline in a modular format, as follows (Figure 1):

REFERENCE—Building the *SpliceWiz* AS reference from genome FASTA and gene annotation GTF files:

1) EXPERIMENT—Dataset processing, which includes the following:
  a) Processing of alignment files to quantify reads mapped to splice junctions, intron coverage metrics and produce coverage files.
  b) Collation of the experiment, which unifies the output data from individual samples, using the splicing annotation as reference, into a self-contained data structure for downstream analysis.
2) ANALYSIS—Loading the collated dataset into memory, (optional) reviewing of sample QC parameters and ASE event filtering, followed by differential analysis using a variety of downstream statistical tools,
3) DISPLAY—Interactive visualization using volcano, scatter and heatmap plots, interactive GO analysis of top differential ASEs, and coverage plots of individual ASEs.

This design allows users with limited programming experience to use the graphical interface to run the full pipeline while enabling those with more bioinformatics experience to automate processes via the command line. Users can apply successive filters on the differential ASEs by directly selecting individual or groups of ASEs from volcano and scatter plots, and/or via gene ontology analysis. Interactively selected ASEs can then be collectively visualized using heatmaps and individually via *SpliceWiz’s* coverage plots.

### Differential analysis

We implemented a GLM-based approach to differential ASE analysis to accommodate large datasets with complex experimental designs, including time series analysis, and accounting for batch effects. Many established statistical methods using GLMs already exist for gene expression analysis, the most cited being *limma* [9], *DESeq2* [10] and *edgeR* [11]. For GLM-based differential AS analysis, we implemented wrapper functions in *SpliceWiz* to utilize each of these statistical tools (see Methods). We also implemented a wrapper function for *DoubleExpSeq* [12], a fast statistical method in R for differential AS analysis using simple contrasts between two conditions (see Methods).

To assess the accuracy of these algorithms at identifying differential ASEs, we simulated reads to emulate transcript expression and technical parameters derived from a real RNA-seq dataset (THP-1 monocyte to macrophage differentiation [13]) using RSEM [14]. We used the percent-spliced-in (PSI) metric to quantify AS, which is determined based on known transcript expression values (see Methods). From these PSI values, a set of ground truth differential events were defined by modelling PSIs using a beta distribution (see Supplementary Methods). Accuracy was measured using two parameters: (i) to assess a method’s ability to accurately detect and appropriately rank the full set of differential events, the area under the receiver operating characteristic curve (AUROC) values were calculated for each method. (ii) To assess precision of the top ranked events in the analysis, we calculated the proportion of ground truth events that overlap with the top K predicted differential events, where K is the number of ground truth differential events in the simulation (hereafter Top-K accuracy).

**Figure 2:**
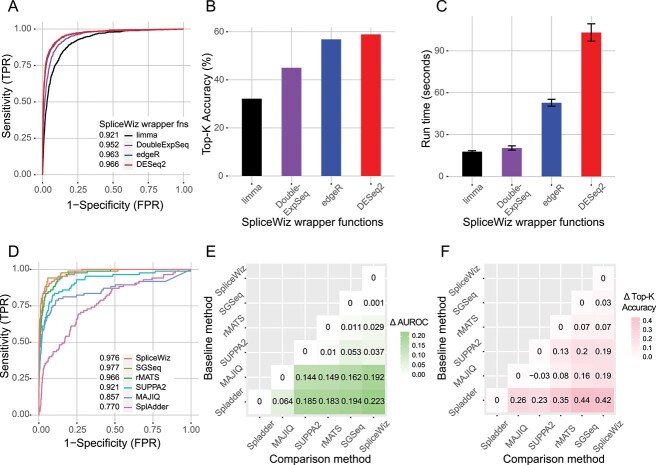
Differential AS analysis benchmarks using the Green *et al*. simulated dataset. (**A**) Receiver operating characteristic (ROC) curves of various SpliceWiz statistical wrapper functions for AS analysis. Numbers indicate AUROC values of each method. (**B**) Top-K accuracy values of various SpliceWiz statistical wrapper functions. (**C**) Mean run-times of each SpliceWiz statistical wrapper, as performed on the simulated dataset (~149 k input events, 3 replicates, 2 conditions). Each method was run in triplicates. Error bars indicate standard deviation. (**D**) ROC curves of SpliceWiz-DESeq2 method and other AS tools, tested against ASEs common to all methods. Numbers indicate AUROC values of each method. (**E**, **F**) Differences in AUROC and Top-K accuracy, respectively, in pairwise comparisons between methods (comparison minus baseline). Events common to each pair of methods are tested in each pairwise comparison benchmark.

We first compared the performance of each statistical wrapper implemented in *SpliceWiz* (Table 1). We found that *DESeq2* and *edgeR* were the top performers, with *DESeq2* marginally better than *edgeR* in both AUROC and Top-K accuracy (Figure 2A, B). Among the remaining methods, *DoubleExpSeq* performed better than *limma*. We also benchmarked the run-time of each method (in analysing the simulation with an input query of 148 633 ASEs for two conditions each with three replicates) and found that *limma* and *DoubleExpSeq* were the fastest methods, whereas *DESeq2* was the slowest (Figure 2C). From this, we concluded that each method serves different purposes. The *limma* wrapper is a useful screening tool in an initial analysis for hypothesis generation. The fast compute time of the *limma* wrapper is especially helpful in large complex datasets where exploratory analysis is performed to identify parameters of interest and identify putative batch factors. The *DoubleExpSeq* wrapper is a general-purpose fast algorithm for simple contrasts between two conditions in a univariate setting. For analyses where an accurate set of top differential ASEs is the highest priority, *edgeR* or *DESeq2* are equivalent options.

**Table 1:** Performance of *SpliceWiz* statistical wrapper functions

*SpliceWiz* wrapper	Run Time (s)	AUROC	Top-K accuracy (%)
*limma*	17.8	0.921	32.1
*DoubleExpSeq*	20.4	0.952	45.0
*edgeR*	52.8	0.963	56.8
*DESeq2*	103.0	0.966	58.9

Next, we compared *SpliceWiz* (using the *DESeq2* wrapper) against other differential AS analysis tools, namely *rMATS* [15], *SUPPA2* [16], *MAJIQ* [17], *SGSeq* [18] and *SplAdder* [19]*.* We chose these tools as they also use the PSI metric. As each method annotates ASEs differently, we calculated AUROC and top-K accuracy values using ASEs commonly annotated by all methods (Figures 2D, S2A). This benchmark identified *SGSeq* as the best performer followed closely by *SpliceWiz*. Additionally, we performed pairwise comparisons using ASEs common to each pair of methods. In pairwise comparison, *SpliceWiz* performed marginally better than *SGSeq*; otherwise the rankings of the other tools did not change with regard to the above metrics (Figures 2E, F, S2B).

Taken together, we conclude that our implementation of GLM-based differential splicing performs well compared with other tools that perform differential AS analysis based on PSI values.

### Coverage plot normalization for intron retention

Current implementations of coverage and sashimi plots are limited in showing coverage of individual samples, rather than mean coverage across replicates of each experimental condition. Combining data from replicates requires a normalization parameter. As replicates differ by both sequencing depth and gene expression, a sample-specific normalization factor is required for each gene. Moreover, variations in depth of coverage due to GC-bias and 3′- (or 5′-) bias mean that, even within the same gene, different normalization factors may be required across each genomic region.

**Figure 3:**
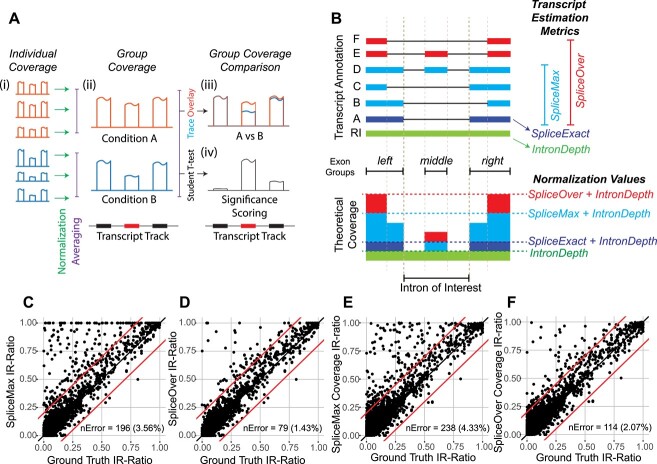
Coverage plot normalization for IR. (**A**) Schematic showing coverage normalization (i) is required prior to combining replicates by group to produce mean normalized coverage plots per condition (ii). Traces of mean coverage can be stacked to accentuate differences in mean coverage (iii), and group differences in normalized coverage can be measured using two-tailed Student’s *t*-test, which can be converted into a trace following negative log-10 transformation (iv). (B) (Top) Example transcript annotation showing various junction read-based spliced transcript estimation metrics (SpliceExact—junctions must exactly span the intron of interest; SpliceMax—junctions can share either coordinate with the intron of interest; and SpliceOver—junctions arise from either of the two flanking exon groups). (Bottom) Schematic of theoretical raw coverage attributed to expression of transcripts as color-coded in the transcript annotation above. Normalization levels based on each transcript estimation metric are represented as horizontal lines that, after normalization, will be converted into unity (i.e. normalized coverage = 1). (**C**, **D**) Scatter plots showing estimated versus ground-truth IR-ratio values based on spliced transcript estimation using SpliceMax and SpliceOver, respectively. (**E**, **F**) Coverage-inferred IR-ratio based on mean coverage across the intron compared against ground truth IR-ratio, based on splice abundance using SpliceMax and SpliceOver, respectively. For (C)–(F), diagonal red lines represent 20% PSI error boundaries and nError refers to the number and percentage of events with >20% PSI error.

However, unlike in *DEXSeq* [20] where a statistical model is used to fit every exon, we sought a method to calculate a single normalization parameter for each ASE, which may overlap across multiple exons and introns. After normalization, the mean coverage across alternate regions should reflect their calculated PSIs, whereas coverage across constitutive regions (i.e. regions expressed by both included and excluded isoforms) should be normalized to unity. Within-group coverage means and variances can then be visualized as lines and shaded ribbons, respectively. Differences in normalized coverage can be statistically tested (Figure 3A).

First, we approached the case of IR and considered the simple scenario where the expressed isoform either involves splicing or retention of the intron of interest (i.e. only transcripts [RI, A] are expressed—see Figure 3B). In this case, coverage normalization should result in the exon boundaries to be anchored to unity and the average coverage across the intron should reflect the IR-ratio, as given by equation (1):


(1) \begin{equation*} IRratio=\frac{Intronic\ Abundance}{Intronic\ Abundance+ Spliced\ Isoform\ Abundance} \end{equation*}

In *IRFinder* [21], intronic abundance is measured using the trimmed mean of intronic coverage of sequencing reads (hereafter *IntronDepth*), which we adopted in *SpliceWiz*. For introns flanked by constitutively spliced exons, the read counts mapping to the splice junction across the intron of interest (hereafter *SpliceExact*) approximates spliced isoform abundance. However, in many cases, at least one exon flanking the intron of interest is also differentially spliced (e.g. due to expression of transcripts [B, C, D, E, F]—Figure 3B). To account for this, *IRFinder* estimates spliced isoform abundance using the *SpliceMax* metric [21], which is calculated as follows: junction reads utilizing each of the splice junctions flanking the intron of interest are first calculated; *SpliceMax* is then determined as the larger of these two values (Figure 3B). In the example illustrated in Figure 3(B), the SpliceMax metric accounts for total splicing where one of the transcripts [A, B, C, D] is the major isoform.

However, we observed that over-estimation of IR-ratios arises in cases where neither splice site bounding the intron of interest is shared with that of the major isoform (e.g. due to expression of transcripts [E,F] in Figure 3B). We encompassed these cases by proposing the *SpliceOver* metric which we define as follows: First, we use the transcriptome annotations to determine exon groups which are genomic regions occupied by mutually overlapping exons. Then, for the intron of interest, we quantified all splice sites that arise from the two exon groups flanking the intron of interest, rather than simply utilizing the flanking splice junction coordinates. *SpliceOver* is then determined as the larger of these two values.

We hypothesized that transcript abundance (the denominator value of the IR-ratio) is best estimated using the sum of *SpliceOver* + *IntronDepth*. We compared this method against that using *SpliceMax* + *IntronDepth* which is the denominator value in calculating IR-ratios in *IRFinder* [21]. Using the simulated RNA-seq dataset based on THP-1 differentiation, we determined ground truth IR-ratios using known transcript abundances (see Supplementary Methods). First, we show we could reproduce *SpliceMax*-based IR-ratios in *SpliceWiz* by comparing these with values produced by *IRFinder-S* [22] (Figure S3A). Next, we compared IR-ratios derived from either *SpliceOver* or *SpliceMax* against ground truth. Using replicate 1 from the THP1-M0 macrophage (simulated) sample as an example, using *SpliceOver* to estimate spliced isoform abundance resulted in less events with IR-ratio error (absolute difference between predicted and actual IR-ratios) is greater than 0.2, compared with *SpliceMax* (1.43% versus 3.56%, Figure 3C,D). We attributed this to the overestimation (IR-ratio error > 0.2) of *SpliceMax* in 1.69% of introns compared with *SpliceOver* (Figure S3B). Consequently, *SpliceOver*, as a metric for spliced isoform abundance, more accurately estimates IR-ratios compared to *SpliceMax* (IR-ratio accuracy area under the ROC curve (AUC) of 98.73 versus 98.21, Figure S3C). We repeated this analysis on all samples in the simulation and found similar results (Figure S3D, E). Moreover, based on the simulated dataset, *SpliceWiz’s SpliceOver* metric outperformed *S-IRFinder* in detecting differential IR events (Figure S3F).

If transcript abundance is used as the normalization parameter for coverage, then the mean normalized coverage across introns is equivalent to their IR-ratios. Thus, we compared mean normalized coverage with their ground truth IR-ratios, using *SpliceOver* and *SpliceMax* as estimates of spliced isoform abundance. We found that *SpliceOver*-based normalization outperformed *SpliceMax* at normalizing coverages (2.07 and 4.33% of introns where IR-ratios inferred from mean coverage was outside 0.2 difference with ground truth (Figure 3E, F), resulting in higher accuracy (IR-ratio error AUC 98.41 and 97.85, respectively, Figure S3G).

Taken together, we implemented coverage normalization using a normalization parameter based on transcript depth across the intron of interest, whereby splicing abundance is measured using the *SpliceOver* metric as described above. Using this approach, normalized coverage across introns accurately reflects their IR-ratios.

### Differences in coverage across alternately spliced regions demonstrate differential splicing

**Figure 4:**
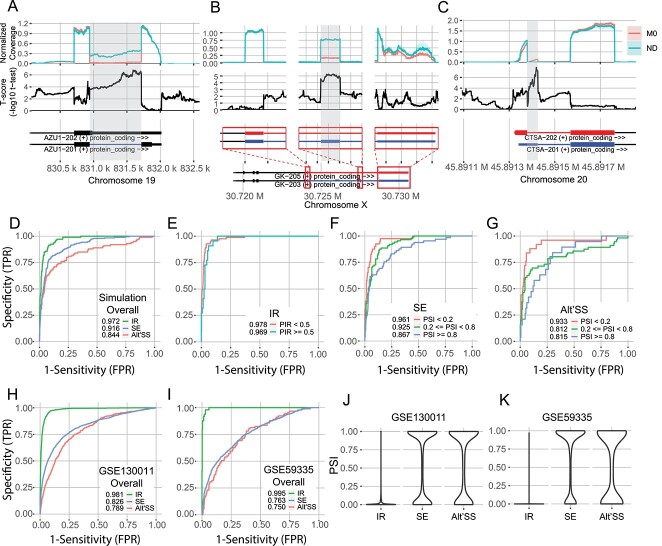
Differences in normalized coverage visualize differential AS. (**A**, **B**, **C**) SpliceWiz group-mean coverage plots (top track); T-scores (−log10 transformed Student *t*-test *P*-values on normalized coverage, middle track), and transcript annotation (bottom track). Areas shaded in grey denote the genomic region of AS (where mean T-scores are calculated). Events shown are (A) IR in AZU1; (B) exon skipping in GK; and (C) alternative 5′-splice site usage in CTSA. (**D**) ROC curve of the T-score that represents the ability of mean normalized coverage plots to visualize splicing, evaluated on the simulated dataset, stratified by the type of ASE. (**E**–**G**) ROC curves of IR, SE and alternative 5′/3′ splice site (Alt’SS) events, respectively, of the T-score on the simulated dataset. Results are stratified by event mean PSIs, using a threshold of 50% for IR events (as there were few events with high PSIs) and 20/80% for other events. (**H**, **I**) ROC curve of T-score evaluated on THP-1 monocyte to macrophage differentiation dataset (Green et al.) and TRA2A/TRA2B dual knockdown in MDA-MB-231 cells (Best et al.), respectively. SpliceWiz edgeR was used to determine differential events. In all ROC curves, numbers in the legend represent AUROC values. (**J**, **K**) Violin plot of PSI value distribution by type of AS in the THP-1 (Green *et al*.) and TRA2A/TRA2B knockdown (Best *et al*.) datasets, respectively.

Having shown that our normalization approach results in mean normalized coverage across introns representing IR-ratios, we examined whether differences in normalized coverage can be used to demonstrate differential splicing between groups of samples. To measure differences in normalized coverages between two groups of replicates, we used a two-tailed Student’s *t*-test on normalized coverage (examples in Figure 4AC). After *P*-values were computed for each nucleotide, we calculated the mean of negative log-10 transformed *P*-values across each alternately spliced region (excluding five boundary nucleotides at each end). We performed this assessment for IR, cassette/ SE and alternate 5′/3′ splice site (A5SS/A3SS) events, whereby the alternatively spliced regions are the introns, SE and the regions between the two alternate splice sites, respectively. We excluded MXE, AFE and ALE events from the analysis as these types of events involve inclusion of two mutually exclusive exons. We also removed from the analysis A5SS/A3SS events where the two alternative splice sites were separated by an intron belonging to an interfering annotated transcript. We evaluated the mean negative log-10 transformed *P*-values from the Student’s *t*-test (hereafter referred to as the T-score) as a discriminator to detect differential events.

We first benchmarked this approach using our simulated dataset, using all ground truth differential ASEs (with ΔPSI >0.05) and nine times as many randomly chosen non-differential ASEs, such that there are 10% differential events. We found that the T-score performed well and was best for IR, followed by SE and then A5SS/A3SS (Figure 4D). Importantly, we found this test was superior for events in which the mean PSI was low (i.e. the alternately spliced region is predominantly excluded across all samples) than those in which the mean PSI was high (Figure 4E–G). Interestingly, when we controlled for PSI by subsetting events using the criterion PSI < 0.1 (Figure S4A, B), there were only negligible differences in accuracy pertaining to different types of ASE (Figure S4C). This suggests that the PSI is the major determinant of accuracy when using coverage to visualize differential splicing.

Next, we benchmarked this approach using two real datasets. We chose the THP-1 monocyte to M0-macrophage differentiation by Green *et al.* ([13], GSE130011) as an example of deep RNA sequencing (~100 million 150-nt paired end reads per sample) and the dual TRA2A/TRA2B knockdown in MDA-MB-231 cells by Best *et al.* ([23], GSE59335) as an example RNA sequencing dataset of a more typical depth (~25 million 100-nt paired end reads per sample). As ground truth differential ASEs cannot be experimentally determined transcriptome-wide, we instead used *SpliceWiz* to perform edgeR-based differential AS analysis as comparison. Events with a false-discovery rate FDR < 0.05 and ΔPSI >0.05 were considered differential ASEs.

First, we addressed whether *SpliceWiz*-based reanalysis could replicate prior findings. The Green *et al.* dataset is known to contain many IR events downregulated in monocyte–macrophage transition [13], whereas the Best *et al.* dataset shows widespread SE in dual TRA2A/TRA2B knockdown [23]. Reanalysis of the Green *et al.* dataset identified 866 and 129 down-regulated and upregulated IR events, respectively, during monocyte–macrophage differentiation. In the Best *et al.* dataset, 821 and 80 differentially skipped and included exons, respectively, due to dual TRA2A/TRA2B knockdown were identified. Thus, in both datasets, *SpliceWiz* reanalysis confirmed prior findings.

Next, we measured the accuracy of *SpliceWiz’s* coverage plots. Again, we benchmarked IR, SE, and A5SS/A3SS differential ASEs and randomly chosen non-differential ASEs such that there are 10% true positive differential events. We observed similar findings, specifically, that the test was more useful for IR than for other types of ASEs (Figure 4H, I), and that this approach was more accurate at low PSI (Figure S4D–H). From this, we concluded that the difference in the performance of coverage plot-based differential splicing visualization between types of AS is largely due to the distribution of PSIs for each type of ASE, (Figure 4J, K). Furthermore, using down-sampling of reads in the THP-1 (Green *et al*.) dataset, we showed that lower sequencing depth led to lower accuracy (Figure S4I–K), which could explain the slightly better accuracy in the Green *et al.* dataset compared with the Best *et al.* dataset.

Taken together, we conclude that group-mean coverage plots are most accurate at demonstrating differential IR but are also useful for demonstrating other forms of AS. Importantly, coverage plots are more accurate at visualizing differential splicing where alternatively spliced regions are predominantly excluded (i.e. at low PSI).

### Interactive exploration of differential analysis

Without tools to interact with the results of differential analysis, identifying top functionally relevant candidates for experimental validation is a manual process that is cumbersome and inefficient. To streamline exploratory analysis downstream to differential analysis, we implemented a series of interactive visualization tools that allow users to identify a set of relevant ASEs following differential analysis.

**Figure 5:**
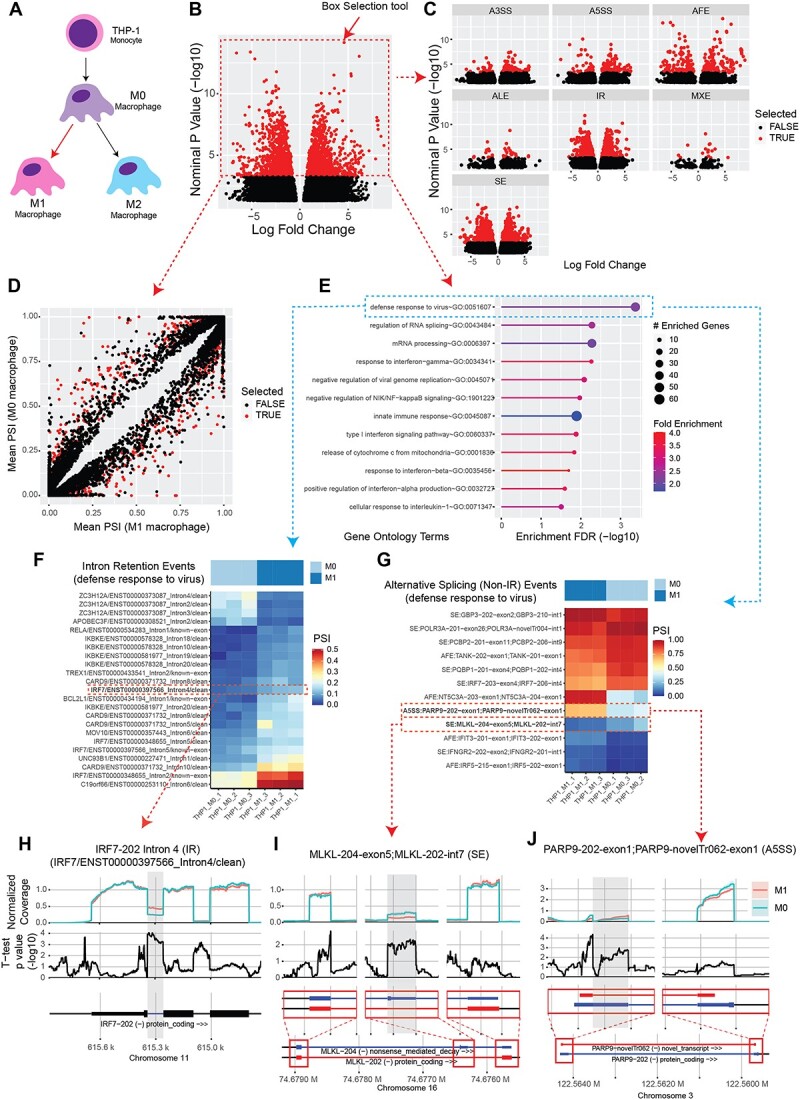
Case study demonstrating SpliceWiz’s interactive data exploration workflow. (**A**) Schematic showing the experimental design of the THP-1 monocyte to macrophage differentiation dataset by Green et al. The following workflow performs differential AS between M0 and M1 macrophage samples. (**B**) Volcano plot of differential ASEs. The user has selected the top events using the box-selection tool using the mouse (as highlighted). (**C**) Volcano plot stratified by type of ASE. (**D**) Scatter plot of mean PSI values between M0 and M1 macrophages. Note that events highlighted in the first volcano plot remained highlighted in red across visualizations. (**E**) Gene ontology enrichment analysis of highlighted ASEs as selected in (B). (**F**, **G**) Heatmap of top IR and other ASEs, respectively, of genes that belong to the top gene ontology category: ‘defence response to virus’. (**H**, **I**, **J**) Coverage plots of example IR (H), SE (I) and alternative 5′ splice site (J) events identified in the previous heatmaps.

As a case study, we use M1 macrophage differentiation from naïve M0 macrophages to illustrate the features (Figure 5A). After differential ASE analysis was performed in contrasting samples from M0 and M1 macrophage differentiation, top differential events can be visualized using interactive volcano plots (Figure 5B, C) or by scatter plots (Figure 5D). Using the mouse, users can highlight ASEs on either plot using box or lasso select tools. In our example, selecting the top events in the volcano plot (Figure 5B) leads to highlighting of the same events in the split volcano plot (Figure 5C) and scatter plot (Figure 5D). Following this, highlighted ASEs can be used to perform GO enrichment analysis (Figure 5E), using as background either the entire set of genes from the annotation, or genes hosting one or more ASEs. Heatmaps can be generated either from top differential ASEs by significance, user-selected ASEs, or ASEs from a GO term (Figure 5F, G). From the heatmap, users can identify ASEs of interest and visualize these as coverage plots (Figure 5H, I, J).

Thus, interactive data exploration downstream to differential analysis is a core feature of *SpliceWiz*, in contrast to currently available tools. Identifying the top functionally relevant ASEs for experimental validation and further study is a key crossroad in any study involving high-throughput AS analysis. Our GUI-based implementation streamlines this process and helps researchers pick the best candidate ASEs without requiring external tools or manual annotation.

### Performance optimization in SpliceWiz

Apart from alignment of raw sequencing reads, the processing of alignment BAM files is the most time-consuming step of AS analysis pipelines. In *SpliceWiz*, BAM files are parsed to count reads aligned across splice junctions, calculate coverage depth across introns, and record coverage for generating coverage plots. Although coverage can be retrieved directly from parsing of coordinate-sorted BAM files, these files are very large and not suitable for transfer between collaborators. As such, an extra step is required to retrieve and compress coverage data as a separate file (traditionally, as BigWig files). Thus, we identified the parsing of alignment files and the storage/recall of coverage data as the two key areas that required performance optimization.

In R, the *htslib* C library [24] (provided via the *Rhtslib* package) is the key developer tool for creating C or C++ − based functions (callable via R) to parse BAM files. Although multi-threaded BAM parsing is supported, downstream access of decompressed data is not thread safe (i.e. reads cannot be processed in a multi-threaded manner). Thus, computationally intensive downstream processing of alignments is not thread-scalable. To overcome this, we developed *ompBAM,* a novel C++ library (provided by the *ompBAM* R package available via *Bioconductor*). Using this resource, developers can create *Rcpp*-based functions to parse and analyse alignment BAM files using multiple threads. Unlike *htslib*, *ompBAM* decompresses alignments which can be later accessed in a thread-safe manner. This allows computationally intensive functions to be thread scalable as downstream processing is no longer bottlenecked at a single thread. *SpliceWiz* uses *ompBAM* for all functions that require parsing of BAM files.

**Figure 6:**
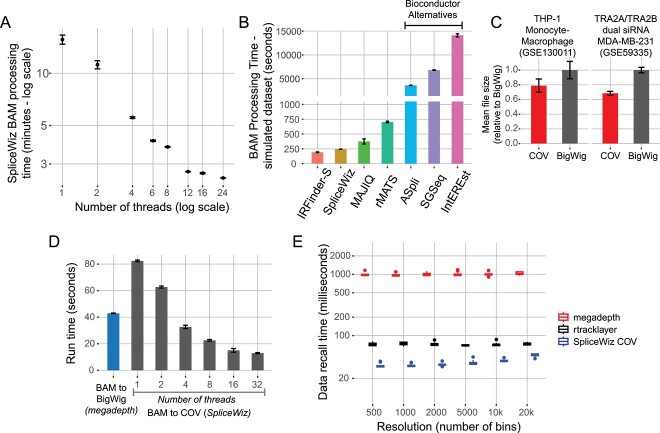
Computational benchmarks of SpliceWiz. (**A**) Time required for SpliceWiz to process BAM files from the simulated dataset, using increasing numbers of threads. Error bars indicate standard deviation of three replicate runs. (**B**) BAM processing time of simulated dataset of various tools, using six threads (IRFinder-S was multi-threaded using BiocParallel via R). Error bars indicate standard deviation of three replicate runs. (**C**) Mean file size of COV files in comparison to BigWig files in the THP-1 (Green *et al*.) and TRA2A/TRA2B dual knockdown (Best *et al*.) datasets. Error bars indicate standard deviation across all samples. (**D**) Time required to generate COV and BigWig files from the THP1-M0 replicate 1 from the THP-1 (Green *et al*.) dataset (~100 million paired-end reads). BigWig files were generated using megadepth. Error bars indicate standard deviation of three replicate runs. (**E**) Box-plot showing time required to retrieve coverage and calculate mean coverage across genomic bins, varied by number of bins (akin to plot resolution). Benchmark was performed using 10 randomly selected long (>50 k nucleotide) genes, each in triplicates.

To demonstrate the performance of *SpliceWiz*, we processed alignments from the simulated dataset using an incremental number of threads and showed that runtime decreased with increasing number of threads indicating thread-scalability (Figures 6A, S5A), with only modest increase in memory consumption (Figure S5B) thereby improving per-thread memory consumption (Figure S5C). Next, we benchmarked *SpliceWiz* against the BAM-processing step from a range of AS tools. Using the same number of threads, *SpliceWiz* outperformed most of the other tools tested, except for *IRFinder-S* (Figure 6B), (noting that *IRFinder-S* does not natively support multi-threading and was parallelized using *BiocParallel* via R). Importantly, *SpliceWiz* is orders of magnitude faster than other AS tools on R/Bioconductor (namely, *ASpli* [25], *SGSeq* [18] and *IntEREst* [26]).

Next, we needed a fast method to store and recall stranded coverage data. As *BigWig* [27] (the current standard for storing coverage data) only supports storage of a single vector of data, each sample would require two *BigWig* files to store stranded coverage data. Thus, we designed a new COV format, which is a BGZF-compressed self-indexed format that stores coverage data for both positive, negative and unstranded data (see Supplementary Methods). Using two datasets, we demonstrate that the COV format shows storage efficiency gains over *BigWig* (Figure 6C). COV file generation is thread-scalable, and at 4+ threads, it is faster than *megadepth* [28], the current fastest tool for generating *BigWig* files (Figure 6D). Importantly, in the default workflow for *SpliceWiz*, COV files are generated simultaneously with BAM parsing to quantify splice metrics, such that BAM files are processed only once (Figure S5D). Data recall using COV files is also faster than equivalent functions that use *BigWig* files. *SpliceWiz* retrieves binned-average coverage faster than R-based tools that utilize *BigWig*, namely, *megadepth* [28] and *rtracklayer* [29] (Figure 5E). This increased performance is due to the greatly improved raw data retrieval time using *SpliceWiz’s* unique COV format (Figure S5E).

Taken together, we successfully optimized the performance of *SpliceWiz*, focusing on alignment file parsing and coverage data storage and recall times. These improvements make *SpliceWiz* suitable for analysing large datasets and generating coverage plots in real time during interactive exploratory analyses.

### Scalability of *SpliceWiz* to large datasets

The computational optimization in *SpliceWiz* allows scalability of R-based differential splicing analysis to large datasets. Additionally, for complex datasets with three or more experimental groups, users may wish to perform differential analysis multiple times to test splicing differences between each pair of groups. In both *rMATS* and *MAJIQ*, performing each comparison requires the re-parsing of intermediate output files for every sample, leading to redundancies in computation. *SpliceWiz* addresses this via its collation step (Figure 1), whereby intermediate output files for each sample are parsed once, unified into count matrices, and compressed into output files along with an abridged version of the splicing reference. Differential analysis is performed by importing the collated dataset from file, rather than by parsing intermediate files of every sample. This design allows *SpliceWiz* datasets to be portable and easy to share among researchers.

**Figure 7:**
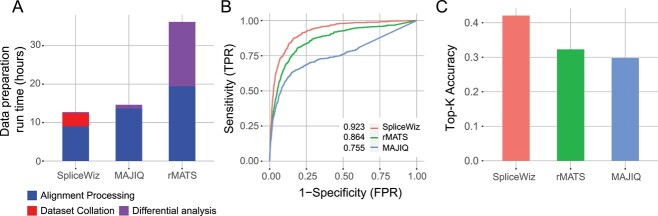
Performance of SpliceWiz in large datasets. (**A**) Total run time to analyse the Leucegene dataset (263 samples). (B) Receiver-operator characteristic (ROC) curves of SpliceWiz-DoubleExpSeq method For Peer Review dataset.

To demonstrate the scalability of *SpliceWiz* and the utility of its modular design to reduce the run time of the downstream differential AS analysis, we analysed 263 acute myeloid leukemia (AML) RNA-seq samples from the Leucegene dataset [30]. For each of the tools (*SpliceWiz*, *rMATS*, *MAJIQ*), we used a maximum of eight threads. Additionally, for *SpliceWiz* and *MAJIQ*, we restricted the available memory to 32 gigabytes. We benchmarked run times for each phase (processing of alignment BAM files, *SpliceWiz* dataset collation and differential analysis between two groups) using the full dataset (263 samples). This benchmark showed that *SpliceWiz* is the fastest tool for both processing of alignment files and subsequent differential analysis (Figure 7A, Table 2). Even when taking to account the extra step of dataset collation, *SpliceWiz* remains the fastest tool.

**Table 2:** Benchmark results using the Leucegene real and simulated datasets

Tool	Alignment processing (hours)	Dataset collation (hours)	Differential analysis (hours)	AUROC	Top-K accuracy
	Leucegene (real dataset, *n* = 263)			Leucegene (simulated dataset, *n* = 20)	
*SpliceWiz*	9.03	3.41	0.29	0.923	0.42
*MAJIQ*	13.7	N/A	0.97	0.755	0.30
*rMATS*	19.4	N/A	16.7	0.864	0.32

Next, we wanted to compare the accuracy of differential analysis in a larger dataset. We assessed accuracy of differential splicing analysis using a simulated dataset based on a subset of the Leucegene dataset (10 acute promyelocytic leukemia samples and 10 randomly chosen AML controls). Accuracy metrics showed that *SpliceWiz* outperformed both *rMATS* and *MAJIQ* (Figure 7B, C, Table 2).

Taken together, *SpliceWiz* scales well to large datasets with respect to computational performance and accuracy. The modular design of *SpliceWiz* facilitates sharing of datasets among collaborators and increases the efficiency for users wishing to perform multiple comparisons in datasets with complex experimental designs.

## Discussion

We demonstrated *SpliceWiz* as a versatile R package for AS analysis. Benchmarks against comparison tools show that *SpliceWiz* is accurate and computationally efficient. Furthermore, *SpliceWiz* implements a novel AS event-based coverage normalization to generate group coverage plots to accurately visualize differential splicing across conditions. Through the *SpliceWiz* GUI, we have also implemented an interactive visualization-based pipeline to enhance the user experience and assist in identifying functionally relevant ASEs for further study.

There are many approaches to benchmarking AS tools to assess accuracy. A key aspect is the assumption of how PSI values are statistically distributed. Our assumption that PSI values are beta distributed is based on the intuition that during sequencing, reads are being randomly sampled from cDNA fragments derived from source RNA that belong to either ‘included’ or ‘excluded’ isoforms. Hence, our benchmarks naturally favour tools that make similar statistical assumptions, namely *rMATS* [15] and *SGSeq* [18]. In contrast, benchmarks that showed *SUPPA2* and *MAJIQ* outperforming *rMATS* were performed based on error models applied to changes in PSI (ΔPSI) as a linear quantity [16, 17]. Accordingly, *SUPPA2* and *MAJIQ* performed less favourably on our benchmarks. Importantly, focussing on PSI as a linear difference between two conditions result in the loss of information about the baseline PSI. When the baseline PSI is close to zero, changes in PSI (e.g. PSI from 0.01 to 0.03, or from 0.99 to 0.97) results in a higher fold-change in logit transformed PSI compared to examples where the baseline PSI is in the center of the [0,1] interval (e.g. PSI 0.49–> 0.51). Thus, like *rMATS* and *SGSeq, SpliceWiz’s* statistical approach is sensitive in detecting ASEs with baseline PSIs close to 0 or 1, which in practice encompasses most ASEs in real datasets (Figure 4J, K).

Prior studies have attempted to combine sample replicates into a single plot. *ggsashimi* [31] combines replicates by stacking traces of raw coverage, using different levels of transparency to show multiple histograms. This approach is limited in its ability to adjust for variances in sequencing depth and gene expressions between replicates, which vary independently of changes in PSI. *Manananggal* [32] implemented an exon-based visualization using a *DEXSeq’s* exon normalization backend, and more recently *VALERIE* [33] implemented an exon-based visualization used a PSI-based normalization which scales coverage based on sum included/excluded junction reads. These approaches are limited in their inability to visualize IR due to their exon-only approach.

Our benchmarks demonstrate that our proposed *SpliceOver* normalization metric estimates local transcript depth to accurately visualize IR. Moreover, group differences in normalized coverage across alternatively spliced regions accurately visualize differential splicing. Importantly, coverage plots are poor at identifying differential events at PSI close to 1. We surmise that this is because coverage plots are asymmetric in their quantitative representation of included and excluded isoforms. Included isoforms are represented by coverage, whereas excluded isoforms are represented only using numbered arcs in sashimi plots. At a PSI close to one, fold change signals in the isoforms are represented as loss of coverage and is hidden among the noise of coverage variances of the included isoform. This finding uncovers a general weakness of coverage plots, which is unrelated to our normalization approach. Naturally, as most IR events have a PSI close to zero whereas other types of ASEs have a bimodal distribution of PSIs around the 0 and 1 boundaries, coverage plots most accurately visualize IR. Nevertheless, coverage plots remain a useful visualization of AS due to its representation of the raw alignment data underpinning splicing quantitation. As larger RNA sequencing datasets become more available, the prior approach of representing a sample group using duplicate/triplicate samples will be replaced by *SpliceWiz’s* approach of representing the entire group via normalized coverage. Thus, normalized coverage plots will become the new standard for AS visualization.

The *SpliceWiz* GUI provides an interactive platform allowing users to explore the results of differential ASE analysis. This platform is designed to highlight interesting and relevant ASEs for further investigation. The choice of ASEs to experimentally verify typically depends on several factors, including the reliability of the measurements and the relevance of affected genes to the functional pathways of interest. Measurement reliability can be visually surveyed using *SpliceWiz’s* normalized coverage plots. Moreover, GO enrichment analysis and GO-based heatmaps facilitate the identification of functionally relevant ASEs. These tools represent a considerable improvement over current methods, which typically provide tabular lists of the top differential results without companion tools or capabilities for functional annotation. Taken together, the interactivity in exploring the differential analysis results improves the user experience and the efficacy in identifying functionally relevant ‘top hits’ for experimental validation.

Computational performance is a key aspect to the user experience of any bioinformatics tool. We optimized *SpliceWiz* to process BAM files orders of magnitude faster than other Bioconductor tools for AS analysis, and on par with command-line tools. Moreover, coverage data stored in *SpliceWiz’s COV* format is more efficient than *BigWig*, which facilitates data transfer between collaborators. These optimizations encourage researchers to analyse AS alongside gene expression, and provide biologists and bioinformaticians with the same platform, thereby enhancing collaboration and communication.

AS databases, whereby AS quantitation has been performed on large RNA-seq datasets in advance, are in growing demand. Recently, numerous AS databases have been published, covering various biological systems including diverse human and murine tissues [34, 35], cancers [36–38] and COVID-19 [39]. *SpliceWiz* provides an ideal platform to create AS databases due to its ability to uniformly analyse and package data from large datasets. *SpliceWiz*-generated AS databases are superior in that it can be used in conjunction with coverage data (via COV files) to visualize differential AS between customizable sample groups. This feature would add to the level of granularity that is required to identify the most meaningful AS events and examine them in the appropriate context. We anticipate that *SpliceWiz* will become the preferred platform for publishing AS database resources going forward.

Although *SpliceWiz* is primarily designed for bulk RNA-seq datasets, alignments from individual or clusters of single cell RNA sequencing (scRNA-seq) can theoretically be analysed using *SpliceWiz*. However, like most AS tools designed for bulk RNA-seq, their use in parsing scRNA-seq is limited by their reliance on full-transcriptome protocols. As most scRNA-seq datasets use 3′-based sequencing, most AS events cannot be properly analysed or visualized. Another limitation of *SpliceWiz* is its inability to correct for technical confounders of sequencing coverage (such as GC and 5′/3′ bias) in its coverage visualization, as *SpliceWiz* only uses a single normalization factor per sample for each ASE. Although batch-correction is a feature implemented in *SpliceWiz* via its GLM-based differential analysis, batch correction cannot be factored into its coverage normalization algorithm.

In summary, *SpliceWiz* is a fast, accessible and user-friendly interactive tool for AS analysis, and sets a new standard in splicing visualization via group normalized coverage plots.

## Methods

### Quantifying percent-spliced-in

ASEs, including annotated IR events, are quantified in *SpliceWiz* using PSI, which is calculated using equation (2). ASE isoforms are designated by the size of the alternately spliced region, which is defined to be larger in the included isoform compared with the excluded isoform. For events where alternatively spliced regions are mutually exclusive (namely MXE, AFE and ALE), the included isoform is defined as that which contains the shorter first intron. For details on annotation of ASEs, see Supplementary Methods and Figure S1: 


(2) \begin{equation*} PSI=\frac{Included}{Included+ Excluded} \end{equation*}

Included and excluded isoform abundances are estimated based on counts of junction reads, noting that such reads do not require length normalization unlike expression estimation using reads aligned to exon bodies. The exception is the included isoform in IR events (i.e. the retained intron), whereby we followed the approach of *IRFinder* [21] in using the (30% trimmed) mean depth of coverage across the measured intron. For isoforms that encompass two (tandem) splice junctions (i.e. included cassette exons or mutually exclusive exons), isoform abundances were estimated using mean counts of the two junctions.

### Differential ASE analysis using established statistical tools in R

Functions for performing GLM-based differential ASE analysis were implemented using wrapper functions to established statistical methods *limma* [9], *DESeq2* [10] and *edgeR* [11]. These were implemented as follows: At the data collation step, two matrices are compiled, each containing estimated included and excluded isoform abundances. Columns in these matrices represent samples whereas rows represent ASEs. During differential expression analysis, two separate analyses are performed. First, differential analysis is performed separately on each of library-normalized included and excluded counts. Assuming a contrasting condition ‘Treatment’ and a batch factor condition ‘Batch’, the model is described in equation (3), where the last term is the contrasting term. This step provides information on whether the change in AS is due to changes in either included or excluded isoform abundances, or both: 


(3) \begin{equation*} design\sim 0+ Batch+ Treatment \end{equation*}

Subsequently, the two matrices (of raw counts) are concatenated column-wise, and an extra ‘ASE’ condition is added to the column (sample) annotations which specifies whether each column represents included or excluded isoforms. Differential ASE analysis is performed using a GLM model as described in equation (4), where again the last term is the contrasting term. This step assesses changes in the ratios of included and excluded counts: 


(4) \begin{equation*} design\sim 0+ Batch+ Treatment+ Treatment: ASE \end{equation*}

We also implemented a wrapper using *DoubleExpSeq* [12], which supports contrasts between two conditions (note that GLM-based models are not supported for the *DoubleExpSeq* wrapper). In *DoubleExpSeq*, the matrices *m* and *Y* are defined as follows: 


$$ m= Included;Y= Included+ Excluded $$

**Key Points**

- *SpliceWiz* incorporates a performance-optimized pipeline with a graphical user interface to provide a user-friendly pipeline for alternative splicing analysis in large, complex datasets.
- A novel normalization technique allows accurate visualization of differential splicing between groups of samples.
- Interactive data exploration aids hypothesis generation by allowing researchers to easily identify functionally relevant candidate alternative splicing events for further study.

## Supplementary Material

Figure_S1_bbad468

Figure_S2_bbad468

Figure_S3_bbad468

Figure_S4_bbad468

Figure_S5_bbad468

Figure_S6_bbad468

SpliceWiz_Table_S1_bbad468

SpliceWiz_Table_S2_bbad468

SpliceWiz_Table_S3_bbad468

SpliceWiz_Table_S4_bbad468

SpliceWiz_Supplementary_Material_Final_v2_bbad468

## Data Availability

*SpliceWiz* and *ompBAM* are available on Bioconductor release 3.17 and above. *SpliceWiz* is also available via: https://github.com/alexchwong/SpliceWiz.

## Author Biographies

**Justin Wong** leads the Epigenetics and RNA Biology Laboratory and is a Senior Lecturer at the Faculty of Health and Medicine, University of Sydney, Australia. His interests include RNA modifications and alternative splicing in normal physiology and disease.

**Alex Wong** is a post-doctoral research officer at the Gene and Stem Cell Therapy Program, Centenary Institute, Australia. His interests include alternative splicing, CRISPR base editing and deep learning in genomics.

**John Rasko** is a professor at the University of Sydney, leads the Gene and Stem Cell Therapy Program, Centenary Institute, and is the Head of Cell and Molecular Therapies at the Royal Prince Alfred hospital, Sydney, Australia. His interests include intron retention, gene and stem cell therapy, experimental haematology and molecular biology.

**Ulf Schmitz** is a bioinformatician and an associate professor at the Department of Biomedical Sciences and Molecular Biology, James Cook University, Townsville, Australia. His interests include systems biology, microRNAs, alternative splicing and intron retention in cancer biology.
