# Supplementary material for: SpliceWiz: interactive analysis and visualization of alternative splicing in R
Source: Brief Bioinform. 2023 Dec 27;25(1):bbad468. doi: 10.1093/bib/bbad468 (PMC10753292; doi:10.1093/bib/bbad468)
Supplement: SpliceWiz_Table_S1_bbad468 [file splicewiz_table_s1_bbad468.docx]

| **Acute Promyelocytic Leukemia** | **Acute Myeloid Leukemia (Control)** |
| --- | --- |
| 01H002 | 03H094 |
| 02H046 | 05H180 |
| 03H070 | 07H060 |
| 04H123 | 09H084 |
| 05H072 | 11H017 |
| 07H043 | 11H103 |
| 07H082 | 12H010 |
| 09H119 | 12H175 |
| 10H053 | 13H018 |
| 12H139 | 14H020 |

**Table S1**: List of samples simulated based on the Leucegene Dataset
