# Supplementary material for: SpliceWiz: interactive analysis and visualization of alternative splicing in R
Source: Brief Bioinform. 2023 Dec 27;25(1):bbad468. doi: 10.1093/bib/bbad468 (PMC10753292; doi:10.1093/bib/bbad468)
Supplement: SpliceWiz_Table_S2_bbad468 [file splicewiz_table_s2_bbad468.docx]

| **Field** | | | **Description** | **Type** | **Value** |
| --- | --- | --- | --- | --- | --- |
| **COV Header**: contains the number, names and lengths of the chromosomes / scaffolds. Analogous to BAM format. BGZF compressed | | | | | |
| magic | | | COV file magic string (equivalent to BAM) | char[4] | COV\1 |
| n_ref | | | # reference sequences | uint32_t | < 2^31^ |
|  | l_name | | Length of the reference name plus 1 (including NUL) | uint32_t |  |
|  | name | | Reference sequence name; NUL-terminated | char[l_name] |  |
|  | l_ref | | Length of the reference sequence, in nucleotides | uint32_t |  |
| **COV Index**: recording chromosome and start coordinate of each BGZF block compressed in the body. BGZF compressed. Positive strand is recorded first, followed by negative, followed by unstranded, thus giving 3 x n_ref number of index blocks | | | | | |
|  | index_size | | Total length of the index entry for each chromosome, excluding this field | uint32_t |  |
|  | | block_start | The first genomic coordinate of the BGZF block | uint32_t |  |
|  |  | file_offset | File offset in of the compressed BGZF block, in bytes, from the beginning of the index block | uint64_t |  |
| **COV Body**: Contains coverage values and lengths in RLE format. The start coordinates of the first value of every uncompressed block is given by the COV index. | | | | | |
|  | BGZF block | | | | |
|  | | cov_value | Depth of RNA-seq coverage | int32_t |  |
|  |  | cov_length | Number of nucleotides with the above coverage value. The start coordinate of this is the end coordinate of the last record. If this is the first record, the start coordinate is given by the block_start value in the index. | uint32_t |  |
| **BGZF end-of-file terminator** | | | | | |

**Table S2**: Overview of the COV format, comprising the header which contains chromosome names and lengths. This is followed by the COV index, which contains (per-chromosome and per-strand) the start coordinates and file offset values of every BGZF compressed block in the body of the file. The COV body contains the coverage data comprising run-length encoded values.
