# Supplementary material for: SpliceWiz: interactive analysis and visualization of alternative splicing in R
Source: Brief Bioinform. 2023 Dec 27;25(1):bbad468. doi: 10.1093/bib/bbad468 (PMC10753292; doi:10.1093/bib/bbad468)
Supplement: SpliceWiz_Table_S3_bbad468 [file splicewiz_table_s3_bbad468.docx]

| **Tool** | **Command / Parameters** |
| --- | --- |
| **(General)** | bams <- SpliceWiz::findBAMS("/path/to/bams", level = 1)  bams_sorted <- SpliceWiz::findBAMS("/path/to/sorted_bams", level = 0) |
| rMATS | rmats_bin <- "/path/to/rmats_turbo_v4_1_1/rmats.py"  system2("/path/to/miniconda3/bin/python", c(  rmats_bin, "--b1", "b1.txt", "--b2", "b2.txt",  "--gtf", "transcripts.gtf", "-t", "paired",  "--readLength", "150", "--variable-read-length",  "--allow-clipping", "--novelSS",  "--nthread", 6,  "--od", rmats_out_path,  "--tmp", file.path(rmats_out_path, "tmp"),  "--task", "prep"  )) |
| MAJIQ | majiq_bin <- "/path/to/env/bin/majiq"  system2(majiq_bin, c(  "build", "transcripts.gff3",  "-c", "majiq_config.txt",  "-j", 6, "-o", majiq_out_path  ))  (inside config.txt)  genome=hg38  readlen=100  strandedness=reverse |
| IRFinder-S | IRF_ref_dir <- "/path/to/IRFinder_reference"  IRF_out_dir <- "/path/to/IRFinder_output"  BPPARAM <- MulticoreParam(6)  bplapply(1:6, function(i) {  dir.create(file.path(IRF_out_dir, bams$sample[i]))  system2("/path/to/IRFinder-2.0-beta/bin/IRFinder", c(  "BAM", "-r", IRF_ref_dir,  "-d", file.path(IRF_out_dir, bams$sample[i]),  bams$path[i]  ))  }, BPPARAM=BPPARAM) |
| IntEREst | out_folder <- "/path/to/interest_output"  # Time to prepare IntEREst reference not benchmarked  int_ref <- readRDS("/path/to/interest_ref.Rds")  system.time({  for(i in seq_len(nrow(bams_sorted))) {  interest(  bamFile = bams_sorted$path[i], isPaired = TRUE,  reference = int_ref,  referenceGeneNames = int_ref$collapsed_gene_id,  referenceIntronExon = int_ref$int_ex,  outFile = file.path(out_folder,  paste0(bams_sorted$sample[i], ".tsv")),  logFile = file.path(out_folder,  paste0(bams_sorted$sample[i], ".log.txt")),  method = c("IntRet", "IntSpan"),  bpparam = MulticoreParam(6)  )  }  }) |
| ASpli | out_folder <- "/path/to/ASpli_output"  targets <- data.frame(  row.names = paste0(rep(c("M0", "ND"), each=3), rep(1:3, 2)),  bam = bams_sorted$path,  Biology = rep(c("M0", "ND"), each=3)  )  # Time to prepare ASpli reference not benchmarked  Features <- readRDS("/path/to/features_ASpli.Rds")  gbcounts <- gbCounts(  features = features,  targets = targets,  minReadLength = 150, maxISize = 50000,  libType="PE",  strandMode=2  )  gbcounts2 <- gbcounts  gbcounts2@junction.counts <- gbcounts2@junction.counts[  substr(rownames(gbcounts2@junction.counts),1,2)!="NA",  ]  asd <- jCounts(  counts = gbcounts,  features = features,  minReadLength = 150,  libType="PE",  strandMode=2  ) |
| SGSeq | # Time to prepare SGSeq reference not benchmarked  txf <- readRDS("/path/to/txf_SGSeq.Rds")  colnames(bams_sorted) <- c("sample_name", "file_bam")  si <- SGSeq::getBamInfo(bams_sorted, cores = 6)  sgfc <- analyzeFeatures(si, features = txf, cores = 6)  sgv <- analyzeVariants(sgfc, cores = 6)  sgv_ranges <- rowRanges(sgv)  sgvc <- getSGVariantCounts(  sgv_ranges, sample_info = si,  cores = 4  ) |

**Table S3**: Pseudo-code describing the commands used to perform run-time performance benchmarks for alignment processing used by the various tools.
