# Supplementary material for: SpliceWiz: interactive analysis and visualization of alternative splicing in R
Source: Brief Bioinform. 2023 Dec 27;25(1):bbad468. doi: 10.1093/bib/bbad468 (PMC10753292; doi:10.1093/bib/bbad468)
Supplement: SpliceWiz_Table_S4_bbad468 [file splicewiz_table_s4_bbad468.docx]

| **Tool** | **Command / Parameters** |
| --- | --- |
| **(General)** | bams_sorted <- SpliceWiz::findBAMS("/path/to/sorted_bams", level = 0) |
| *SpliceWiz* | ## In R:  library(SpliceWiz)  ## For alignment processing  system.time({  processBAM(  bams_sorted$path, bams_sorted$sample,  “/path/to/reference”, “/path/to/pb_folder”,  n_threads = 8  )  })  ## For dataset collation  system.time({  expr <- findSpliceWizOutput(“/path/to/pb_folder”)  collateData(  expr, “/path/to/reference”, “/path/to/nxtse_folder”,  novelSplicing = TRUE, n_threads = 8, lowMemoryMode = TRUE  )  })  ## For differential splicing  system.time({  se <- makeSE(“/path/to/nxtse_folder”)  se <- se[applyFilters(se),]  anno <- fread("conditions.txt")  colData(se)$has_PMLRARa <- as.character(anno$has_PMLRARa[  match(colnames(se), anno$sample)  ])  res <- ASE_DoubleExpSeq(  se, "has_PMLRARa", "TRUE", "FALSE",  IRmode = "annotated_binary”  )  }) |
| *rMATS* | ## NB prep_n.txt files contain 16 BAM files each  ## For alignment processing, the time taken to run the following bash code was benchmarked:  for i in {0..16}  do  rmats.py --b1 prep_${i}.txt --gtf transcripts.gtf \  -t paired --readLength 100 --variable-read-length \  --allow-clipping --novelSS \  --nthread 8 --od rmats_out_prep_8cores \  --tmp rmats_out_prep_8cores/tmp_${i} \  --task prep  done  ## For differential analysis, after output files were copied into the `rmats_out_prep_8cores/tmp_post` directory, the time taken to run the following bash code was benchmarked:  rmats.py --b1 b1.txt --b2 b2.txt --gtf transcripts.gtf \  -t paired --readLength 100 --variable-read-length \  --allow-clipping --novelSS \  --nthread 8 --od rmats_out_prep_8cores \  --tmp rmats_out_prep_8cores/tmp_post \  --task post |
| MAJIQ | ## For alignment processing, the time taken to run the following bash code was benchmarked:  majiq build transcripts.gff3 -c config.txt \  -j 8 -o majiq_out_8cores  ## For differential analysis, the time taken to run the following bash code was benchmarked:  input_path=majiq_out_8cores  [ -d $input_path/PSI_pos ] && rm -rf $input_path/PSI_pos  mkdir $input_path/PSI_pos  [ -d $input_path/PSI_neg ] && rm -rf $input_path/PSI_neg  mkdir $input_path/PSI_neg  [ -d $input_path/dPSI ] && rm -rf $input_path/dPSI  mkdir $input_path/dPSI  s1=`cat majiq_conditions.txt \| grep TRUE \| cut -f 1 -d ','`  s2=`cat majiq_conditions.txt \| grep FALSE \| cut -f 1 -d ','`  majiq psi -j 8 -o $input_path/PSI_pos -n POS --minreads 3 ${s1}  majiq psi -j 8 -o $input_path/PSI_neg -n NEG --minreads 3 ${s2}  majiq deltapsi -j 8 -o $input_path/dPSI -n POS NEG --minreads 3 \  -grp1 ${s1} -grp2 ${s2}  voila tsv -j 8 $input_path/splicegraph.sql $input_path/dPSI/POS-NEG.deltapsi.voila -f $input_path/dPSI/voila_output.tsv \  --non-changing-between-group-dpsi 0.001 --show-all  (inside config.txt)  genome=hg38  readlen=100  strandedness=reverse  (inside majiq_conditions.txt)  A two-column comma-separated table with the first column containing .majiq file paths of each sample, and the second column annotating the PML-RARA fusion status of each sample (TRUE or FALSE) |

**Table S4**: Pseudo-code describing the commands used to perform run-time performance benchmarks for benchmarking performance of processing the Leucegene full dataset (263 samples).
