# Supplementary material for: SpliceWiz: interactive analysis and visualization of alternative splicing in R
Source: Brief Bioinform. 2023 Dec 27;25(1):bbad468. doi: 10.1093/bib/bbad468 (PMC10753292; doi:10.1093/bib/bbad468)
Supplement: SpliceWiz_Supplementary_Material_Final_v2_bbad468 [file splicewiz_supplementary_material_final_v2_bbad468.docx]

**Supplementary Methods**

**Implementation of multi-threaded alignment file processing**

Alignment (BAM) files from RNA sequencing samples typically contain up to 100 million alignment sequences. Processing of these alignments for the purpose of recording alignment coverage requires storage of genomic start and stop coordinates, which consumes a substantial amount of memory (~10 gigabytes for 100 million alignments). Thus, processing multiple BAM files (each using a single thread) is not a viable option as it will result in high memory consumption. To optimally utilize CPU resources while limiting memory consumption, each file would need to be sequentially processed with all available threads.

Although the *htslib* C library [1] provided via the *Rhtslib* R package has already implemented multi-threaded BAM decompression, the alignments are retrieved in sequence and thread-safe analysis of these alignments is not possible (i.e., a “race condition” may arise where different threads are reading from the same alignment). Thus, we implemented routines to decompress BAM files to facilitate downstream multi-threaded analysis. These routines are provided as C++ header files via the *ompBAM* (OpenMP BAM) R package which is available via *Bioconductor*.

*ompBAM* allows R developers to write C/C++ routines for reading and analysing BAM files by simply including the *ompBAM* header files. These provide two objects: the *pbam_in* object, which opens and decompressed BAM files to extract alignments, and the *pbam1_t* object contains functions to interpret alignment data. Data decompressed by *pbam_in* is stored in a memory buffer and can be retrieved in a thread-safe manner for processing by child threads. This buffer is refreshed with more decompressed data only after all alignments have been retrieved, thereby ensuring all alignments are processed.

File decompression occurs after a call to *ompBAM’s* *pbam_in::fillReads()* function and proceeds via the following steps: (i) a portion of compressed data (read into memory from the prior iteration) is analysed to determine BGZF block start positions, which is designated to decompression threads; (ii) each designated thread decompresses their allocated data which is stored in a single pre-allocated decompression buffer; and (iii) decompressed data is analysed to determine the memory locations of each alignment whereby equal-size blocks of alignments are allocated to each thread. During (ii), for N available threads, N-1 threads are allocated to decompression, while the remaining thread is designated to read more compressed data from file into memory.

After the decompression step, R developers can write code for the analysis of alignments. This should be implemented in an OpenMP parallel loop, whereby each thread can retrieve their allocated alignments via *pbam_in::supplyRead()*, which returns a *pbam1_t* object. This function takes a single parameter which is the ID number of each child thread in the parallel loop. All alignments must be processed before the next iteration of *fillReads()* can be called. Alignments that cannot be processed in the current iteration (e.g., unpaired reads) can be stored by “realizing” the alignment via the *pbam1_t::realize()* function. This function makes a copy of the alignment data from the contiguous decompressed memory buffer into a separate location, so the data persists after the next decompression call.

As in *htslib’s* *bam1_t*, *ompBAM’s* *pbam1_t* contains convenience functions to retrieve alignment data, including core data, sequence, cigar, read quality scores, and alignment tags.

**Experimental Datasets**

Three RNA-seq datasets were downloaded from NCBI Gene Expression Omnibus: GSE131101 is a dataset of THP-1 monocyte to macrophage (M0) and subsequent M1/M2 macrophage differentiation (triplicate samples, ~100 million 150-nt paired end reads) by Green *et al* [2]. GSE59335 is a dataset of dual siRNA-based knockdown of TRA2A/TRA2B in MDA-MB-231 cells (with untreated controls, each in triplicate, ~20-25 million 100-nt paired end reads) by Best *et al* [3]. GSE67039 is an RNA-seq dataset of tumour samples from 263 patients with acute myeloid leukemia, which forms part of the Leucegene dataset [4].

**Simulation Datasets**

Two datasets were simulated:

- undifferentiated THP-1 cells and THP-1-derived M0-macrophages from Green *et al* [2] provided a dataset with two conditions and 3 replicates per condition.
- Samples from 10 patients with APML (as evidenced by the presence of the PML-RARA fusion RNA detected using *STAR-Fusion* version 1.7.0 [5]) and 10 randomly-chosen control AML samples from the Leucegene dataset [4] provided a larger dataset of two conditions with 10 replicates per condition (Table S1).

Sequencing reads from samples of each dataset were analysed with *RSEM* [6] version 1.3.3, using the *rsem-calculate-expression* function, to obtain “isoform.results” files which contain transcript expression values in transcripts per million (tpm), and “.model” files which contain model estimates pertaining to the technical characteristics of the RNA-seq dataset. These files were then used as input to generate simulated reads using the *rsem-simulate-reads* function (20 million 150-nt paired-end reads per replicate for the Green et al dataset, and 40 million 100-nt paired end reads per replicate for the Leucegene dataset). The “isoform.results” files generated by the *rsem-simulate-reads* function were used as ground truth transcript expression values.

**Data Preprocessing**

Raw sequencing FASTQ files were extracted from SRA files using the *fasterq-dump* utility of the *sratoolkit* version 11. Alignment of raw sequences was performed using the *STAR* splice-aware aligner version 2.7.3a [7]. Additionally, FASTQ files were analysed using *Salmon* version 1.9.0 [8] to obtain transcript expression values to be parsed using SUPPA2 [9]. For all tools, we used the *Ensembl* GRCh38 human genome and gene annotation (release 94) as input reference.

**Annotation of alternative splicing events**

In *SpliceWiz*, ASEs are annotated by the set of coordinates of splice junctions defining included or excluded isoforms (Figure S1). In principle, *SpliceWiz* defines the included isoform as the one that “includes” a larger proportion of the gene or that contains the shorter upstream intron. Specifically, the included isoform is defined as:

- the included exon (in SE)
- the 5ʹ-most mutually exclusive exon (in MXE)
- the retained intron (in IR)
- the shorter spliced intron (in A5SS, A3SS, AFE, ALE).

To identify SE and MXE events, for each transcript a list of skip junctions (D_i_, A_i+1_) is compiled. D and A denote donor and acceptor splice junction coordinates; i representing the intron number in the transcript; i∈[1,…,n-1] where n is the number of exons in the transcript. SE events were defined by matching a skip junction of the included isoform with an intron of the excluded isoform. MXEs were defined by unique transcripts sharing a common skip junction but containing cassette exons (A_i_, D_i+1_) with different 5ʹ- and 3ʹ-coordinates.

AFEs were defined as transcript pairs with first introns sharing a common acceptor site, whereas ALEs were defined as transcript pairs with last introns sharing a common donor site.

A5SS and A3SS events were defined as transcript pairs sharing introns with common acceptors and donors, respectively. Additionally, we required alternate splice sites to belong to the same exon group. Exon groups were defined by determining the union of the coordinate ranges of mutually overlapping exon groups. In determining these groups, we excluded transcripts with retained introns (i.e., those with transcript_biotype “sense_intronic” or “retained_intron”) as retained introns are annotated as “exons” that span across two or more otherwise-distinct protein-coding exons.

**Defining ground truth differential alternative splicing events in the simulated datasets**

PSI values of ASEs (as defined in the previous section) were determined for each replicate of the simulated datasets. For each ASE, we determined a list of transcripts compatible with either included or excluded isoform. These lists were used to calculate (included and excluded) isoform expression values based on ground truth transcript expressions used to generate the simulation. These were then used to determine PSI (equation 2 of the main text).

For intron retention (IR), we additionally determined ground truth IR-ratios. We defined “spliced transcripts” as the set of transcripts that contain an intron with genomic ranges overlapping that of the intron of interest, whereas “IR-transcripts” were the set of transcripts that contain an “exon” overlapping that of the intron of interest. As per above, we determined isoform expression values of “IR-transcripts” and “spliced transcripts” for each intron (which contains at least 1 annotated “IR-transcript”) to calculate ground truth IR-ratios (equation 1 of the main text).

After determining PSIs, we modelled PSIs using a beta distribution as implemented in *DoubleExpSeq’s* *DBGLM1* function [10]. As input, we used included isoform counts and ASE transcript counts (i.e., included + excluded isoform counts) as *y* and *M* parameters into the *DBGLM1* function, using default parameters (i.e., `shrink_method’=”WEB”` and “fdr.level=0.05”). For the Green *et al* simulated dataset, we defined significance as a Benjamini-Hochberg adjusted p-value (BH-padj) of less than 0.05. For the Leucegene simulated dataset, we used a nominal p value of 0.01 which yielded 480 significant differential ASEs, because the more stringent criterion (BH-padj < 0.05) yielded only 48 significant differential ASEs, which was insufficient for benchmarking purposes. Further, we used an additional test by fitting PSI values using the *gamlss* function from the *gamlss* R package version 5.4-10 [11], using beta distribution model. Because many results had at least 1 sample with PSI values of zero or one, we modelled these distributions using beta zero-inflated, one-inflated, or beta-inflated models for ASEs with at least one PSI-value being 0, 1, or both, respectively. We used the same process to determine ground truth differential IR-ratios. In calculating IR-ratios from ground truth transcript expressions, we considered a *spliced transcript* as any transcript with an intron that overlapped that of the intron of interest along the genomic axis. ASEs with low total transcript expression (sum of included and excluded isoform expression less than 0.1 tpm) were excluded from analysis.

**Benchmarking accuracy of differential alternative splicing tools**

For benchmarking the accuracy of differential ASE analysis on a typical dataset, the Green *et al* simulated dataset by comparing the following tools: *SpliceWiz* version 1.3.0, *rMATS* version turbo v4.1.1 [12], *SUPPA2* version 2.3 [9], *MAJIQ* version 2.4.dev3+g85d07819 [13], *SGSeq* version 1.32.0 [14], and *SplAdder* version 3.03 [15]. We also analysed intron retention using IRFinder-S version 2.0.1 [16]. For benchmarking accuracy using a larger dataset, the Leucegene-based simulated dataset was analysed using *SpliceWiz*, *rMATS* and *MAJIQ*. For *SUPPA2*, we analysed raw FASTQ files using *Salmon* version 1.9.0 [8], for which transcript expression values were used as input to *SUPPA2*. For other tools, we used alignment BAM files as input.

True and false positive rates (for ROC curves) and AUROC values were determined using the *ROCit* R package version 2.1.1. Nominal (raw) p-values provided by each tool were used to rank putative differential ASEs reported by each tool. For *MAJIQ*, we used the probability of no difference in place of p values. To evaluate SE and MXE events in MAJIQ, we paired source and target local splicing variations (LSVs) and used the minimum of the two probabilities of no difference of source and target in place of p-value. For each tool, p-values (multiplied by -1) were used as input for the “score” parameter for the *rocit* function.

Top-K accuracy was calculated by first determining K, the number of true positive ASEs that were tested in each comparison analysis. Top-K accuracy was then calculated by determining the proportion overlap between top K events predicted by each tool and that of true positive events.

For comparison between all six tools (*SpliceWiz*, *SGSeq*, *rMATS*, *SUPPA2*, *MAJIQ* and *SplAdder*), the set of common events annotated by all tools were used to determine AUROC and Top-K accuracy values. For pairwise comparisons, the set of events common to each pair of tools were used (Figure S2B).

**Calculation of the normalization parameter for normalized coverage plots**

*SpliceOver* is a method which estimates spliced transcript abundance based on junction counts involving the exon groups flanking the intron of interest. It is used to estimate local transcript depth, which is the sum of *SpliceOver* and *IntronDepth* (the latter is the 30% trimmed mean depth of coverage across the intron). Local transcript depth is the parameter used to normalize coverage for respective ASEs.

For each intron, *SpliceOver* is the larger of the two values *SpliceOverLeft* and *SpliceOverRight*, which are the sum junction counts involving the “left” and “right” flanking exon groups (with the genomic axis orientated left-to-right). For A5SS / A3SS / AFE / ALE events, the larger of the local transcript depths of the two alternate introns is used. For SE and MXE events, local transcript depth values are calculated for each junction, for which the largest local transcript depth is used.

**Benchmarking accuracy of *SpliceWiz* coverage plots**

*SpliceWiz* generates a “T-test track” by performing Student’s t-tests on normalized coverages between two groups of samples, for each nucleotide along the genomic track, followed by negative log-10 transformation. To test the utility of this track in discriminating between differential and non-differential alternatively spliced regions, for each ASE, we calculated its mean values across the differentially spliced region. For IR events, this is the intronic region; for SE this is the cassette exon, and for alternative splice sites this is the part of the exon between the two splice sites.

Benchmarking was performed on both the Green *et al* simulated dataset, and two real datasets (Green *et al* and Best *et al* datasets). Ground truth differential ASEs for the simulated dataset was obtained as described above, with the additional criterion requiring ΔPSI > 0.05). For real datasets, transcriptome-wide experimentally determined ground truth values are not available. Hence, we used the differential ASEs obtained using *SpliceWiz*’s differential analysis results (edgeR wrapper), with the additional criterion requiring ΔPSI > 0.05, for calculation of the ROC curves.

**COV file implementation for storing RNA-seq coverage data**

The current implementation of *BigWig* is not optimized for stranded RNA-seq coverage data, which requires the handling of positive, negative and unstranded coverage values on the same genomic axis. To enable streamlined read/write access of stranded coverage data, we designed the COV file format (Figure S6, Table S2). COV is a binary indexed file format utilizing BGZF-based compression, allowing random read access as in *BigWig*. However, COV accommodates 3 vectors of coverage data for positive, negative and unstranded RNA-seq coverage. Every BGZF block (64 kb) of compressed coverage data is indexed by the genomic start coordinates of the data it contains and its file offsets, allowing for fast random-access data retrieval. COV files are generated concurrently with junction and intronic read quantitation in the *SpliceWiz* pipeline, resulting in minimal additional runtime and resource overhead. These optimizations allow *SpliceWiz* to seamlessly store and recall the data required to produce coverage plots on-demand.

**Benchmarking computational performance of alignment processing**

Performance of multi-threaded *SpliceWiz* using incremental numbers of threads was benchmarked on a high-performance cluster (HPC) allowing us to enforce the number of available threads. Computational performance of alignment processing was benchmarked using the 6 alignment files from the simulation dataset (each containing ~20 million paired end alignments). Comparison tools were run using 6 available threads. For *IRFinder-S* [16], where multi-threading is not implemented, we used *BiocParallel* to process BAM files in parallel (one file per thread). *ASpli* [17], *SGSeq* and *IntEREst* [18] were run on a high-performance cluster, whereas *rMATS*, *MAJIQ* and *IRFinder-S* were run on a Linux server. Using 6 threads on the simulated dataset, *SpliceWiz* performed equally on both HPC and local Linux server (mean run time of 244.9 and 244.5 seconds, respectively).

For benchmarking run times of *SpliceWiz*, *rMATS* and *MAJIQ* using the Leucegene dataset, the required tools were installed using a conda environment on the HPC server. Benchmarks were performed once, where run times of alignment processing, dataset collation and differential analysis were measured. Except for *rMATS*, benchmarks were performed using a maximum of 8 threads and 32 gigabytes of memory.

Run times were measured either using the *microbenchmark* R package version 1.4.9, using the *system.time()* function in base R, or by scrutinising the “Walltime used” field in the “Job Summary” output of queued jobs on the HPC server. Memory consumption was measured using `/usr/bin/time -v` prepended to the executed function in the command line. All benchmarks were performed in triplicate unless otherwise specified. Commands used to benchmark BAM processing time of the simulated Green *et al* and native Leucegene datasets are shown in Tables S3 and S4, respectively.

**Benchmarking computational performance of COV (coverage) file format**

To measure storage efficacy, *COV* files were produced from BAM files from the two experimental datasets. Positive and negative stranded coverage data were retrieved via *SpliceWiz* in R and exported as *BigWig* files using the import function in the *rtracklayer* R package version 1.58.0 [19]. Size comparisons were performed against *COV* files and corresponding stranded *BigWig* files (sum of file sizes of positive and negative strand coverage). *COV* generation time using incremental threads was benchmarked on a HPC, using *SpliceWiz’s* *BAM2COV* function, and using *processBAM* with or without *COV* output (configured via the ‘*skipCOVfile’* argument), and was compared to *megadepth* R package version 1.0.3 [20], the latter of which does not support multi-threading.

Two methods of data retrieval were benchmarked. To emulate retrieval of data for plotting, average coverage of genomic interval bins was measured, as each bin represents a pixel on a plot and the number of bins is the horizontal resolution. We normalized gene counts using *limma’s voom* function [20] to identify 10 randomly chosen highly expressed genes in the simulation. For each gene, the genomic interval was divided into equally sized “bins”, whereby the average coverage was calculated for each bin. Raw data retrieval (shown in Figure 6E) was benchmarked using *SpliceWiz’s getCoverage function* and *rtracklayer’s import* function (BigWig).

**Supplementary Tables**

Table_S1

Table_S2

Table_S3

Table_S4

**Supplementary Figures**

Figure_S1

Figure_S2

Figure_S3

Figure_S4

Figure_S5

Figure_S6

**Supplementary References**

1. Bonfield JK, Marshall J, Danecek P, Li H, Ohan V, Whitwham A, et al. HTSlib: C library for reading/writing high-throughput sequencing data. Gigascience. 2021;10(2).

2. Green ID, Pinello N, Song R, Lee Q, Halstead JM, Kwok CT, et al. Macrophage development and activation involve coordinated intron retention in key inflammatory regulators. Nucleic Acids Res. 2020;48(12):6513-29.

3. Best A, James K, Dalgliesh C, Hong E, Kheirolahi-Kouhestani M, Curk T, et al. Human Tra2 proteins jointly control a CHEK1 splicing switch among alternative and constitutive target exons. Nat Commun. 2014;5:4760.

4. Lavallée VP, Baccelli I, Krosl J, Wilhelm B, Barabé F, Gendron P, et al. The transcriptomic landscape and directed chemical interrogation of MLL-rearranged acute myeloid leukemias. Nat Genet. 2015;47(9):1030-7.

5. Haas BJ, Dobin A, Li B, Stransky N, Pochet N, Regev A. Accuracy assessment of fusion transcript detection via read-mapping and de novo fusion transcript assembly-based methods. Genome Biology. 2019;20(1):213.

6. Li B, Dewey CN. RSEM: accurate transcript quantification from RNA-Seq data with or without a reference genome. BMC Bioinformatics. 2011;12(1):323.

7. Dobin A, Davis CA, Schlesinger F, Drenkow J, Zaleski C, Jha S, et al. STAR: ultrafast universal RNA-seq aligner. Bioinformatics. 2013;29(1):15-21.

8. Patro R, Duggal G, Love MI, Irizarry RA, Kingsford C. Salmon provides fast and bias-aware quantification of transcript expression. Nat Methods. 2017;14(4):417-9.

9. Trincado JL, Entizne JC, Hysenaj G, Singh B, Skalic M, Elliott DJ, et al. SUPPA2: fast, accurate, and uncertainty-aware differential splicing analysis across multiple conditions. Genome Biology. 2018;19(1):40.

10. Ruddy S, Johnson M, Purdom E. Shrinkage of dispersion parameters in the binomial family, with application to differential exon skipping. The Annals of Applied Statistics. 2016;10(2):690-725.

11. Rigby RA, Stasinopoulos DM. Generalized additive models for location, scale and shape. Journal of the Royal Statistical Society Series C: Applied Statistics. 2005;54(3):507-54.

12. Shen S, Park JW, Lu Z-x, Lin L, Henry MD, Wu YN, et al. rMATS: Robust and flexible detection of differential alternative splicing from replicate RNA-Seq data. Proceedings of the National Academy of Sciences. 2014;111(51):E5593.

13. Vaquero-Garcia J, Barrera A, Gazzara MR, González-Vallinas J, Lahens NF, Hogenesch JB, et al. A new view of transcriptome complexity and regulation through the lens of local splicing variations. Elife. 2016;5:e11752.

14. Goldstein LD, Cao Y, Pau G, Lawrence M, Wu TD, Seshagiri S, et al. Prediction and quantification of splice events from RNA-Seq data. PLoS One. 2016;11(5):e0156132.

15. Kahles A, Ong CS, Zhong Y, Rätsch G. SplAdder: identification, quantification and testing of alternative splicing events from RNA-Seq data. Bioinformatics. 2016;32(12):1840-7.

16. Lorenzi C, Barriere S, Arnold K, Luco RF, Oldfield AJ, Ritchie W. IRFinder-S: a comprehensive suite to discover and explore intron retention. Genome Biology. 2021;22(1):307.

17. Mancini E, Rabinovich A, Iserte J, Yanovsky M, Chernomoretz A. ASpli: integrative analysis of splicing landscapes through RNA-Seq assays. Bioinformatics. 2021;37(17):2609-16.

18. Oghabian A, Greco D, Frilander MJ. IntEREst: intron-exon retention estimator. BMC Bioinformatics. 2018;19(1):130.

19. Lawrence M, Gentleman R, Carey V. rtracklayer: an R package for interfacing with genome browsers. Bioinformatics. 2009;25(14):1841-2.

20. Wilks C, Ahmed O, Baker DN, Zhang D, Collado-Torres L, Langmead B. Megadepth: efficient coverage quantification for BigWigs and BAMs. Bioinformatics. 2021;37(18):3014-6.
